# Urogenital *Chlamydia trachomatis* multilocus sequence types and genovar distribution in chlamydia infected patients in a multi-ethnic region of Saratov, Russia

**DOI:** 10.1371/journal.pone.0195386

**Published:** 2018-04-11

**Authors:** Valentina A. Feodorova, Svetlana S. Konnova, Yury V. Saltykov, Sergey S. Zaitsev, Irina A. Subbotina, Tatiana I. Polyanina, Sergey S. Ulyanov, Susanna L. Lamers, Charlotte A. Gaydos, Thomas C. Quinn, Vladimir L. Motin

**Affiliations:** 1 Laboratory for Molecular Biology of Chlamydia, Federal Research Center for Virology and Microbiology (FRCViM), Pokrov, Vladimir region, Russia; 2 Laboratory for Molecular Biology and NanoBiotechnology, Federal Research Center for Virology and Microbiology (FRCViM), Branch in Saratov, Saratov, Russia; 3 Department for Microbiology, Biotechnology and Chemistry, Saratov State Agrarian University (SSAU), Saratov, Russia; 4 Department for Medical Physics, Saratov State University (SSU), Saratov, Russia; 5 Bioinfoexperts LLC, Thibodaux, Los Angeles, United States of America; 6 Division of Infectious Diseases, Johns Hopkins University School of Medicine (JHUScM), Baltimore, Maryland, United States of America; 7 Division of Intramural Research, National Institute of Allergy and Infectious Diseases (NIAID), Baltimore, Maryland, United States of America; 8 Department of Pathology, Department of Microbiology & Immunology, University of Texas Medical Branch (UTMB), Galveston, Texas, United States of America; Rudjer Boskovic Institute, CROATIA

## Abstract

**Background:**

This is the first report to characterize the prevalence and genovar distribution of genital chlamydial infections among random heterosexual patients in the multi-ethnic Saratov Region, located in Southeast Russia.

**Methods:**

Sixty-one clinical samples (cervical or urethral swabs) collected from a random cohort of 856 patients (7.1%) were *C*. *trachomatis* (CT) positive in commercial nucleic acid amplification tests (NAATs) and duplex TaqMan PCRs.

**Results:**

Sequence analysis of the VDII region of the *ompA* gene revealed seven genovars of *C*. *trachomatis* in PCR-positive patients. The overall genovars were distributed as E (41.9%), G (21.6%), F (13.5%), K (9.5%), D (6.8%), J (4.1%), and H (2.7%). CT-positive samples were from males (n = 12, 19.7%), females (n = 42, 68.8%), and anonymous (n = 7, 11.5%) patients, with an age range of 19 to 45 years (average 26.4), including 12 different ethnic groups representative of this region. Most patients were infected with a single genovar (82%), while 18% were co-infected with either two or three genovars. The 1156 bp-fragment of the *ompA* gene was sequenced in 46 samples to determine single nucleotide polymorphisms (SNP) among isolates. SNP-based subtyping and phylogenetic reconstruction revealed the presence of 13 variants of the *ompA* gene, such as E (E1, E2, E6), G (G1, G2, G3, G5), F1, K, D (D1, Da2), J1, and H2. Differing genovar distribution was identified among urban (E>G>F) and rural (E>K) populations, and in Slavic (E>G>D) and non-Slavic (E>G>K) ethnic groups. Multilocus sequence typing (MLST) determined five sequences types (STs), such as ST4 (56%, 95% confidence interval, CI, 70.0 to 41.3), ST6 (10%, 95% CI 21.8 to 3.3), ST9 (22%, 95% CI 35.9 to 11.5), ST10 (2%, 95% CI 10.7 to 0.05) and ST38 (10%, 95% CI 21.8 to 3.3). Thus, the most common STs were ST4 and ST9.

**Conclusion:**

*C*. *trachomatis* is a significant cause of morbidity among random heterosexual patients with genital chlamydial infections in the Saratov Region. Further studies should extend this investigation by describing trends in a larger population, both inside and outside of the Saratov Region to clarify some aspects for the actual application of *C*. *trachomatis* genotype analysis for disease control.

## Introduction

*Chlamydia trachomatis* (CT) is one of the most commonly occurring sexually transmitted infections (STIs) in both young men and females with an annual estimate of 105.7 million new cases worldwide [1]. Because of its high epidemic potential, undiagnosed and untreated Chlamydial infection can result in a number of complications. The World Health Organization (WHO) estimates the cost-burden for treating chlamydia patients, especially among adolescents, is approximately $10 billion annually [2]. During the past two decades, several countries worldwide have focused studies on genotyping local CT cases to enhance the understanding of clonal diversification, genovar prevalence and evolution, level of transmission, and to address the role of co-infection with two or more CT variants. This knowledge could significantly improve the ability of national and trans-national surveillance programs to build global STI surveillance systems [1].

There is little information on CT genovar distribution in the Russian Federation besides the Moscow and St. Petersburg Regions [3,4,5]. National diagnostic laboratories have developed the capability to molecularly detect CT DNA in clinical specimens of chlamydial patients by highly sensitive nucleic acid amplification tests (NAATs) [6]. However, these detection systems do not provide genetic tools to discriminate CT genovars, even for the *ompA* gene polymorphism that has been generally accepted as a single-locus typing standard [7]. For further discrimination of *C*. *trachomatis* strains between and within genotypes, we additionally applied the multilocus sequence typing (MLST). This procedure is based on the analysis of polymorphism of housekeeping genes, and is widely used for genotyping many microorganisms, including the evaluation of Chlamydia diversity [8–11]. Although the determination of Chlamydia sequences types (STs) and genovars may not be essential for the clinical outcome and treatment schedule, it can certainly shed light on the global epidemiology of this pathogen. Nevertheless, a recent single nucleotide polymorphism (SNP) based study of Smelov et al. [5] revealed the association between the phenotypic diseases (lymphogranuloms venerium, urethritis and cervicitis, and ocular trachoma) and branches in phylogenetic tree. Another important question was whether, as others have previously reported [12–17] there are differences in the genovar’s distribution among different ethnicity groups that may reflect variations in the selection of certain genovars in different parts of the world. The goal of this study was to investigate the prevalence of *C*. *trachomatis* infection among patients in multi-ethnic European Region located in Southeast Russia. The relationships between CT genovar distributions, gender, age, nationality and place of residence of patients were examined. Our research revealed the most common CT sequences types (STs) in the Saratov Region.

## Materials and methods

### Setting

The Saratov Region population (~ 2.6 million people) has an approximately equal percentage of men (45.8%) and women (54.2%), with total of 22 ethnic groups (Slavic population of 87.6% of Russian descent, and non-Slavic ones, such as Kazakhs (3.1%), Tatars (2.2%), Ukrainians (1.7%), Jews (0.1%), Germans (0.3%) and other nationalities (5%). The urban population (~ 1.9 million) is located in a large Southeastern European part of Russia (S = 101200 km^2^), which borders Kazakhstan.

### Clinical samples

Clinical samples (cervical or urethral swabs) from a random cohort of heterosexual patients (n = 856, women (n = 400) and men (n = 400) and anonymous (n = 56), from August, 2011 to January, 2012), who reported to one of seven different diagnostic laboratories of the Saratov Region for detection of chlamydia infection were screened for *C*. *trachomatis* to confirm current infection. Specimens were routinely collected and directly delivered for PCR testing using the Chlamydia Transport-Single Swab (COPAN, Italy). All the patients had symptoms of typical complaints for chlamydial infection, such as lower abdominal pain, pronounced vaginal discharge, frequent urination, post-coital bleeding, inter-menstrual bleeding, and others. Each patient provided written informed consent. This study was approved by the Human Bioethics Committee of the Saratov Scientific and Research Veterinary Institute No. IRB00008288 (http://ohrp.cit.nih.gov/search/IrbDtl.aspx).

### *C*. *trachomatis* detection and typing

Total DNA was isolated from clinical specimens using the DNeasy Blood and Tissue Kit (QIAGEN GmbH, Hilden, Germany) according to the manufacturer’s instructions. DNA samples were routinely analyzed by conventional real-time PCR kits (Central Research Institute of Epidemiology, Moscow, Russia) that targets the presence of the *C*. *trachomatis* cryptic plasmid [6]. The samples were also analyzed by a duplex TaqMan PCR designed to simultaneously detect the cryptic plasmid and the 16S RNA gene of *C*. *trachomatis* (“*C*. *trachomatis*-RT-quantity", SYNTOL, Moscow, Russia). The results were verified by real-time PCR (Vector-Best, Novosibirsk, Russia) targeted to both cryptic plasmid gene and *gyrA* gene [6] coupled to DNA isolation with magnetic silica particles.

The genotyping of *C*. *trachomatis* in the positive samples was performed by amplification and sequencing of the Variable Domain (VDII) of the *ompA* gene. Three forward and four reverse primers, previously designed by Quint *et al*. [18], were used in amplifications with all possible individual primer combinations (12 pairs) for each sample. These generated amplicons of 157 to 160 bp that were sequenced by using the same pair of primers. The consensus sequence of the reads from direct and reverse chains and NCBI Blast (http://blast.ncbi.nlm.nih.gov/Blast.cgi) were used to determine the CT genovar. Subsequently, samples with an identified genovar were used in an amplification reaction with the forward primer F1 (CGGTATTAGTATTTGCCGCTTTG) and B11 [19] that amplified a 1156 bp fragment of nearly full *ompA* gene. The amplicon was sequenced with the use of flanking and internal primers for the *ompA* gene. The subtyping of each genovar was based on the reference strains designated in the study of Lysén *et al*. [12]. The sequences of the *ompA* gene were aligned with the reference strains to identify single nucleotide polymorphisms and their impact on translated sequences.

Primer specificity and protocol validation for basic CT detection was implemented as described [20] using specific relevant DNA from referent Chlamydial strains (CT, *Chlamydia pneumoniae*, *Chlamydia psittaci*, *Chlamydia abortus* etc, kindly gifted by Dr Hasanova TA) in parallel with Cobas Amplicor (conventional PCR) and Cobas TagMan48 (real-time PCRs) (Roche Diagnostics, Branchburg, NJ, USA) approved by FDA [21].

All representative genovar sequences reported in this research were deposited in GenBank (accession numbers KU963174-KU963186). The evolutionary tree was inferred in MEGA 7 using the Neighbor-Joining method with 100 bootstrap replicate samples. ModelTest in MEGA7 was used to identify the most appropriate model of evolution (the Tamura 3-parameter method [22]). All positions containing gaps were eliminated. The tree was drawn using Figtree software (http://tree.bio.ed.ac.uk/software/figtree/).

MLST based on seven housekeeping genes (*gatA*, *oppA*, *hfiX*, *gitA*, *enoA*, *hemN and fumC*) were performed as described [4,8,9]. A consensus sequence was created from forward and reverse sequence reads, genes were concatenated, and queried against MLST sequences in the PubMLST database to find identical allelic profile known as STs (https://pubmlst.org). Multiple sequence alignments of the sequence output were created by ClustalW (http://www.ebi.ac.uk/Tools/msa/clustalw2/). Phylogenetic tree was constructed using the UMPGA hierarchical clustering method in MEGA 7 [22]. Strain clustering and SNP analyses were performed as described [9] to define the relationships between strains at the microevolutionary level.

### Statistical analysis

The geographical, gender, age and ethnic origin data were analyzed using Graphpad Software. Categories of data were presented in the rows of each matrix (genovars D/E/F/G/H/J/K, and STs, ST4/ST6/ST9/ST10/ST38); marginal totals, reflecting the presence or absence of specific genovar and STs, were presented in the columns of the matrix (male/female, Rural/Urban, and Slavic/non-Slavic). Proportions of individuals, who have and do not have the specific genovar and STs inside each of the category, were compared. Results of *ompA* genovar and STs distribution was estimated using 95% confidence interval (95% CI). The significance of the differences in distribution of *ompA* genovar and STs between proportions of mono-infected and multiple-infected individuals was statistically compared by the Chi-square test for categorical data or the Fisher’s exact test when the number of samples was small. A *p*-value<0.05 was considered significant.

## Results

### Baseline subject characteristics enrolled

**Fig 1:**
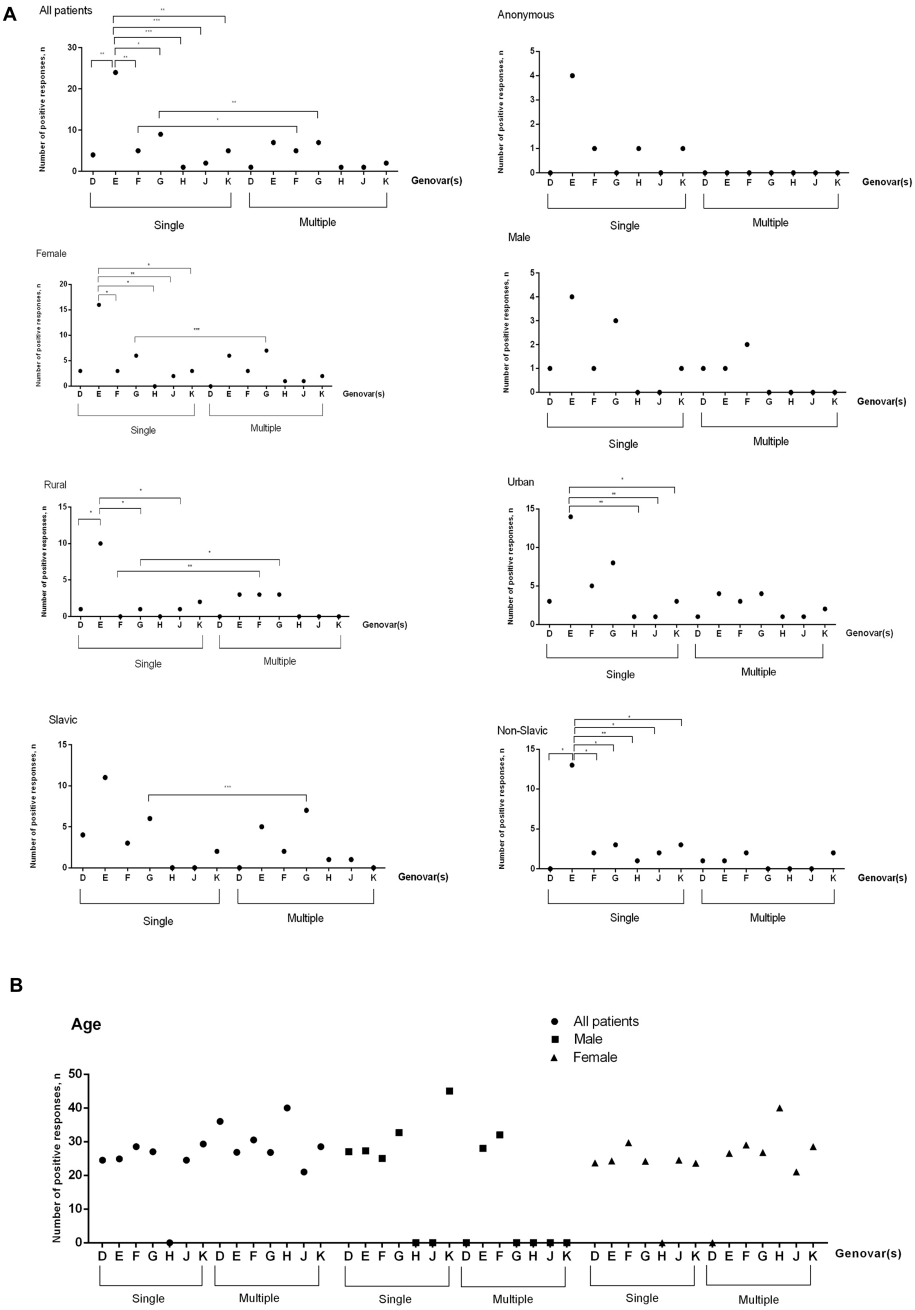
Distribution of genovars in CT-positive patients. **(A) According to their gender, place of residence and ethnic origin. (B) According to their age in mono-infected and multiple-infected CT-positive samples**. Analysis was done by Fisher’s exact test (two-tailed). Statistically significant differences are indicated by * (*p*<0.05) or by ** (*p*<0.01) or by *** (*p*<0.001). Slavic cohort included Russians, Byelorussians and Ukrainians, Non-Slavic cohort was presented by Caucasians, Jews, Kyrgyzs, Koreans, Moldavians, Germans, Mordovians, and anonymous.

*C*. *trachomatis* DNA was detected in 61 of 856 samples tested (7.1%). The CT-positive patients’ ages ranged from 19 to 45 years (average 26.4), with a subset (11.5%) who gave no personal information (anonymous, n = 7 of 61) and 88.5% (54 of 61) who agreed to provide gender, age, place of residence and nationality data (S1 Table). The majority were women (42 of 54, 77.8%), while men contributed fewer samples (12 of 51, 22.2%). Urban citizens (42 of 61, 68.8%) were predominantly infected over the rural residents (19 of 61, 31.2%). At least 12 different ethnic groups were CT-infected (S1 Fig). In total, at least seven *C*. *trachomatis* genovars, D, E, F, G, H, J and K, were identified in the positive patients. The following serovar distribution was observed: E—41.9%, G—21.6%, F—13.5%, K—9.5%, D—6.8%, J—4.1%, and H—2.7% (74 DNA samples were genotyped). The majority of the patients (82%, 50 of 61) were infected with a single genovar, while 18% (11 of 61) demonstrated co-infection with either two or three CT genovars (Fig 1A). The distribution of CT genotypes in patients with monoinfection and among multiple-infected persons were similarly distributed, with E>G>F/K>D>J>H and E>G/F>K>D/H/J, respectively. However, in patients with mono-infection the presence of genovar E only was observed more frequently when compared to all other genovars detected, such as D, F and K (*p*<0.01), G (*p*<0.05) and genovars H and J (*p*<0.001). No statistically significant difference in CT genovar distribution was registered in multiple-infected patients (*p*>0.05). At least two genovars, F (*p*<0.05) and G (*p*<0.01) were found to be present more often among mono-infected and multiple-infected patients (Fig 1A, panel All patients).

Further in the group of infected patients (n = 61) we observed that genovars E and G were the most prevalent mono-infections for almost each category (Fig 1A). In the same category of mono-infected individuals, genovar E was observed more frequently in comparison with genovars F, H, K (*p*<0.05) and J (*p*<0.01) in female, genovars D, G and J (*p*<0.05) in rural, K (*p*<0.05), H & J (*p*<0.01) in urban, H (*p*<0.01) and the rest of genovars (*p*<0.05) in Non-Slavic population. There was no statistically significant difference (*p*>0.05) in distribution of genovars in such groups of mono-infected patients as male, anonymous, and Slavic individuals, as well as in all categories of multiple-infected patients. Among other genovars, genovar G was more often (*p*<0.05) observed in mono-infected than in multiple-infected patients, namely in female (*p*<0.001), rural (*p*<0.05) and Slavic (*p*<0.001) patients (Fig 1A). There was a statistically significant difference in distribution of genovar F in mono-infected and multiple-infected individuals in a single group of rural population (*p*<0.01). Overall, genovars D, F, J and K were identified occasionally in each group. Genovar H was rarely present and detected only in a single anonymous urban patient. The combination of genovars as D+F, E+F, E+G, E+K, F+G, F+K, E+H+G, and E+J+G was seen in the group of multiple-infected patients. The distribution of different genovars was similar in the group of male and female patients (Fig 1B).

### *ompA* subtyping

Analysis of the 1156 bp-fragment of the *ompA* gene, comprising four variable (VD) and five constant (CD) domains, revealed 13 genetic variants of *C*. *trachomatis* in clinical samples of the PCR-positive patients (Table 1). Overall, seventeen substitutions in three variable (VDI, VDII and VDIV) and three constant (CDI, CDIII and CDIV) domains were identified across all genovars when compared to reference strains. Only five (29.4%) nonsynonymous substitutions were seen in the variable regions, such as: VDI—1, VDII—1 and VDIV—3. In contrast, 12 of 17 (70.6%) substitutions were located in constant domains, namely: CDI—9, CDIII—2, and CDIV—1. In fact, three of these 12 (25%) substitutions were silent (synonymous substitutions): 2—in CDI, and 1—in CDIV. The remaining nine (75%) substitutions would induce an amino acid change (non-synonymous substitution). Of the nine observed amino acid mutations, only one mutation significantly altered the amino acid’s general characteristics (Da2, K75E), which mutates a strongly positive amino acid (K) to a strongly negative amino acid (D).

**Table 1:** The *ompA* gene polymorphism found in 13 genetic variants of 61 clinical *C*. *trachomatis* specimens collected in Saratov Region, Russia, compared to reference sequences^a^.

Genotype (no. of cases/total of this subtyped genovar)	MOMP Region	Nucleotide change	Amino acid Change	Strain name (accession number of identical sequences in GenBank)
**D1** (1/3)		Reference strain		D/B120 (X62918)
**Da2** (2/3)	CDI	C132T	G44G	D/IC-Cal8 (X62920)
	CDI	G154A	A52T	
	CDI	G184A^b^ T186G ^b^	V62M ^b^	
	CDI	C195T	Y65Y	
	CDI	A223G	K75E	
	CDI	T228A	T76T	
	CDI	T246C	F82F	
	CDI	G249C	Q83H	
	CDIII	A636T	S212S	
**E1** (18/22)		Reference strain		E/Bour (X52557)
**E2** (3/22)	VDIV	G997A	A333T	E/IU-TC0755ut(ACI43896.1)
**E6** (1/22) ^c^	VDIV	G995A	S332N	DK-K31 (AM901187.1)
**F1** (4/4)		Reference strain		F/IC-Cal3 (X52080)
**G1** (1/6)		Reference strain		G/UW57 (AF063199)
**G2** (1/6)	VDIV	T1003G	S335A	9768 (CP001887.1)
**G3** (3/6)	CDI VDII CDIII VDIV	T228A G487AG700C T1003A	T76T G163S E234Q S335T	I-149 (DQ116400.1)
**G4** (0/6)	VDII	G487A	G163S	G/11222 (CP001888.1)
**G5** (1/6) ^c^	VDII VDIV	G487A T1003A	G163S S335T	This study
**H1** (0/1)		Reference strain		H/UW4 (AF304857.1)
**H2** (1/1)	VDI CDIV	A272G C850T	N91S L284L	ATCC VR-879 (JX564246.1)
**J1** (3/3)		Reference strain		J/UW36 (DQ064292.1)
**K** (7/7)		Reference strain^d^		K/UW31/Cx (AF063204)

^**a**^ The reference strain and subtype definitions were taken from Lysen *et al*. [12]

^**b**^ Two varied positions within the same codon produce a single amino acid difference

^**c**^This subtype is absent in the study of Lysen *et al*. [12] and was numbered by us

^d^ Genovar K has no subtypes

**Fig 2:**
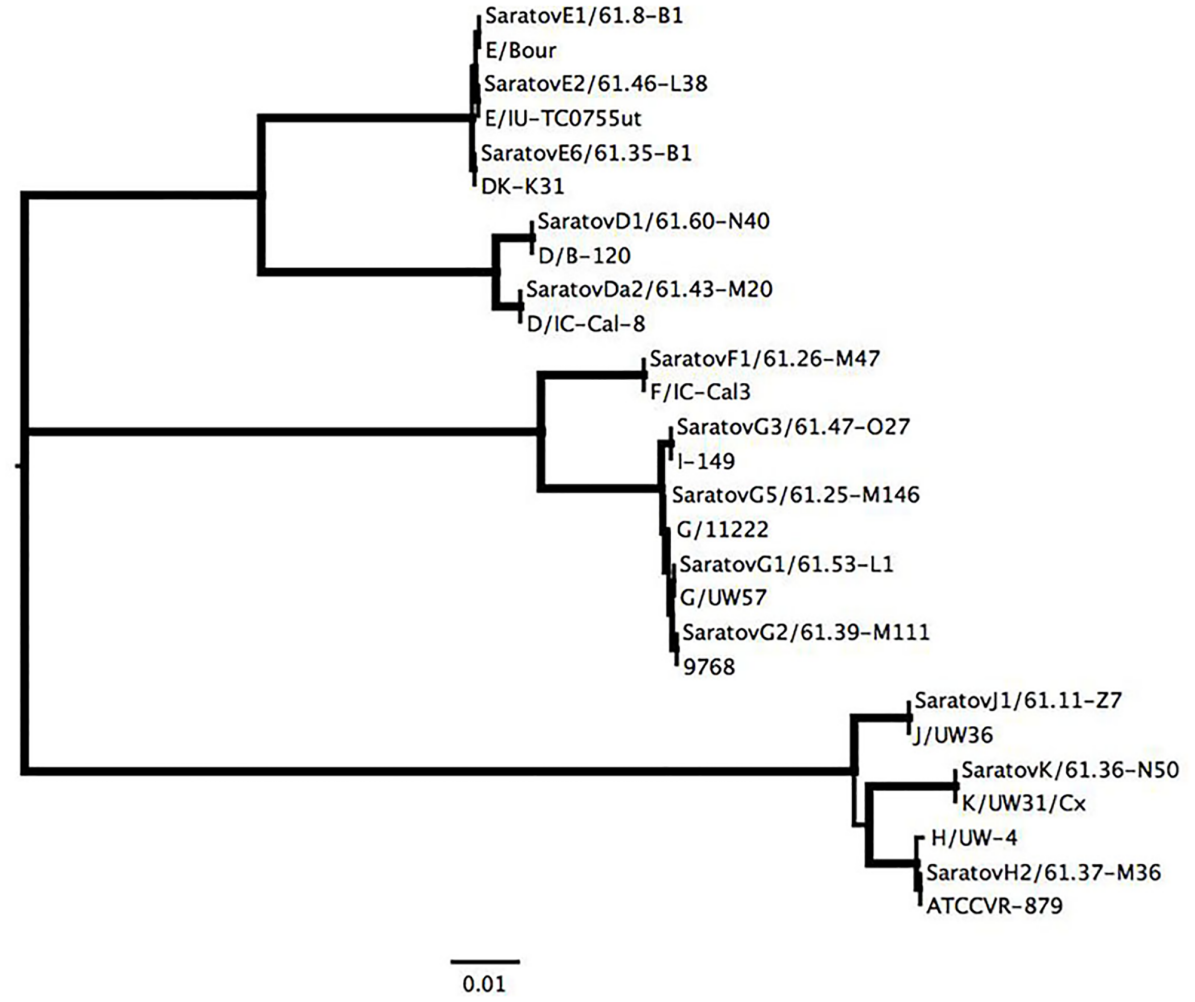
Phylogenetic tree demonstrating the relationship of Saratov *ompA* sequences to references strains from Table 1. Thick branches, subtending all large clades, represent 100% bootstrap support. The scale at the bottom represents one substitution per site.

In the majority of the genovar E sequences, 18 of the 22 subtyped (81.8%) showed higher similarity to the reference strain E/Bour of subtype E1. However, we identified two additional subtypes of the E2 variant reported by Lysen *et al*. [12] with a single point mutation at position 997 (3 of 22 subtyped, 13.6%), and another variant with an SNP at position 995 (1 of 22, 4.6%), which was detected earlier in Europe [7,23]. This subtype was absent in the study by Lysén *et al*. [12] for genovar E, therefore, we designated it as E6 (Table 1). All E1, E2, and E6 sequences could be distiguished by a single SNP, and branched in a significant phylogenetic clade (Fig 2).

Of the 9 specimens with mono-infection with a second predominant genovar G determined by sequencing the short VDII region, we were able to amplify and sequence the large amplicon of the *ompA* gene from 6 samples (Table 1). Lysen *et al*. [12] described the subtypes G1-G4, and we identified three of these in our samples: G1 (1 of 6, 16.7%), G2 (1 of 6, 16.7%) and G3 (3 of 6, 50%). Moreover, we identified a novel subtype G5, which contained a combination of two previously described SNPS that were not present in the current version of GenBank. One SNP was located at position 487 of the VDII region and was observed in subtypes G3 and G4, and another one was positioned at 1003 of VDIV and was detected in G2 and G3 subtypes. Thus, the G5 subtype represents a combination of single SNPs of G2 and G4 variants that could be seen in the corresponding cluster of the genovar G variants on the dendrogram (Fig 2). The subtypes of the other genovars revealed the presence of well-known variants, which are also represented in the reference strains of Lysen *et al*. [12], such as D1, F1, J1 and K (no subtypes known), as well as more rare variants, such as Da2 and H2 (Table 1, Fig 2).

### MLST analysis

**Fig 3:**
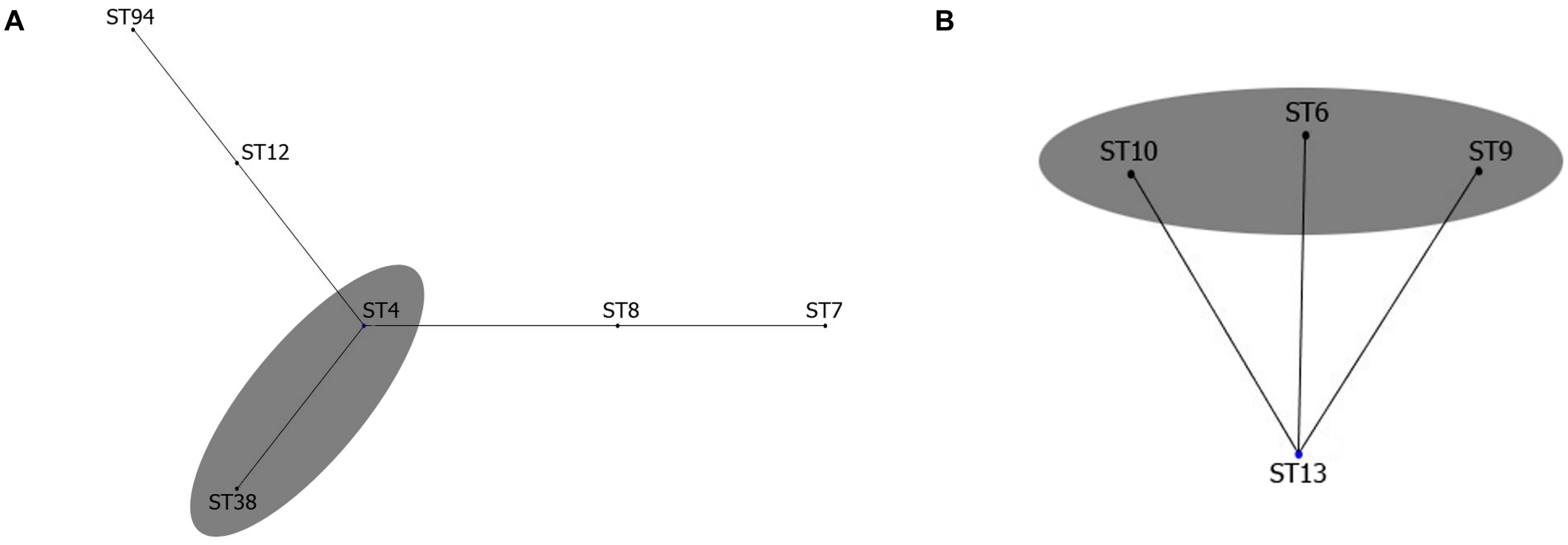
Clonal grouping of sequence types (STs) of *C*. *trachomatis* strains found in our study (marked with gray ovals). The division into Group I (A) and Group III (B) was based on the EBURST analysis with other STs of these clonal complexes obtained from PubMLST/Chlamydiales database.

Five STs were determined by MLST (Table 2) among 13 genetic variants based on the *ompA* genotyping of *C*. *trachomatis* (Table 1). The majority of them (>91%), excluding ST38, were assigned to genetic lineages of two groups, such as Group I (ST6, ST9 and ST10, Fig 3A) and Group III (ST4, Fig 3B). Therefore, our STs could be allocated to two out of three non-overlapping clonal complexes identified early by Pannekoek *et al*. [9] from global collections (1959–2009 years) of urogenital, ocular and rectal strains of *C*. *trachomatis* [24]. The ST38 found by us here was not described in these studies [9,24]; however, this type was found later in Moscow region [4]. The ST38 belongs to Group III as CT strains of ST38 demonstrated identical alleles with six out of the seven loci found in ST4 and differed from latter ST by a single allele *hflX* (Table 2). In fact, both ST38 and ST4 have been found to be the members of a single clonal complex [4] of the relevant Group III in which the ST4 was a putative founder for ST38 [9]. Thus, the Saratov CT strains of ST38 together with those of ST4 were assigned to genetic lineage of Group III (Fig 3B). This rare ST38, which was discovered first in Moscow in 2005 [4], is related to CT isolates found exclusively in Europe (Sweden) in 2002 and in the UK in 2009 [24]. No ST13, the putative founder for Group I [4,9] was revealed in our study (Fig 3A). Almost all STs detected here (ST4, ST6, ST9 and ST38), except ST10, were present among the STs from the samples of the clinical cases in the Moscow region [4].

**Table 2:** The *ompA* and seven housekeeping genes MLST sequence types of Saratov and reference strains of *C*. *trachomatis* obtained from the PubMLST database (https://pubmlst.org).

ST (MLST)	No. of CT strains		Allele							*ompA* genovar(s)	Reference strain(s) information		
	Abs.	%	*gatA*	*oppA*	*hfiX*	*gitA*	*enoA*	*hemN*	*fumC*		Name (accession number of identical sequences in PubMLST database	*ompA* genovar	Reference
**4**	28	56	3	1	1	2	4	2	3	E	E/Sweden2 (269)	E	9,24
			3	1	1	2	4	2	3		SaratovE1/61.8-B1	E	This study
			3	1	1	2	4	2	3	D	D/IC-Cal-8 (7)	D	9
			3	1	1	2	4	2	3		SaratovDa2/61.43-M20	D	This study
**6**	5	10	3	3	2	5	3	1	3	K	K/UW-31 (14)	K	9,24
			3	3	2	5	3	1	3		SaratovK/61.36-N50	K	This study
**9**	11	22	3	3	2	4	3	2	3	G	G/11074 (242)	G	5,24
			3	3	2	4	3	2	3		SaratovG1/61.53-L1	G	This study
			3	3	2	4	3	2	3	J	G/IOL-238 (16)	J	9
			3	3	2	4	3	2	3		SaratovJ1/61.30-A32	J	This study
**10**	1	2	3	3	2	1	3	2	3	H	H/UW-4 (18)	H	5,9,24
			3	3	2	1	3	2	3		SaratovH2/61.37-M36	H	This study
**38**	5	10	3	1	2	2	4	2	3	F	F/SW4 (239)	F	5,24
			3	1	2	2	4	2	3		SaratovF/61.26-M47	F	This study
**94**	0	0	3	4	1	32	4	2	3	E	E/Bour (235)	E	5,24

**Fig 4:**
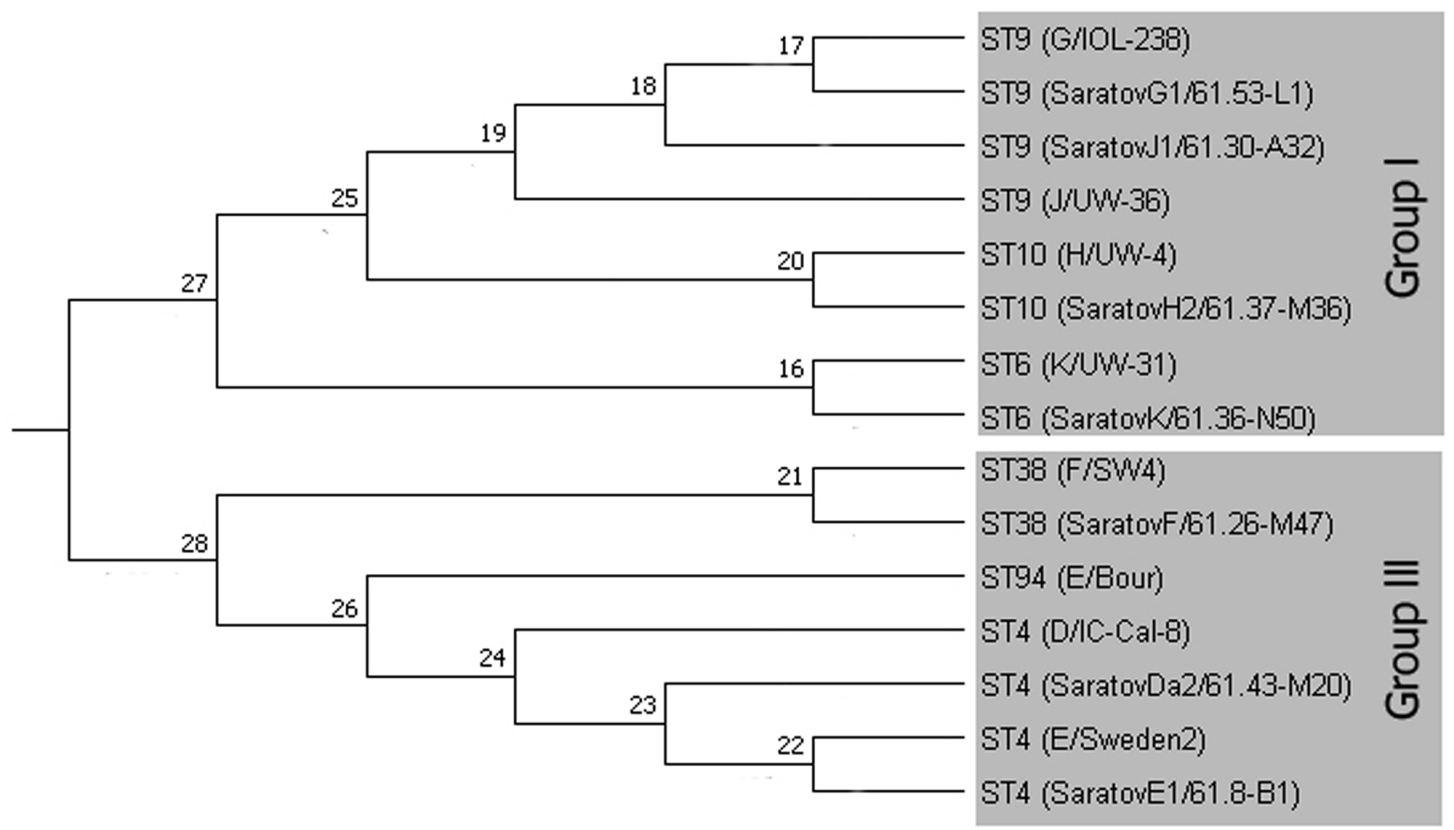
Phylogenetic analyses of concatenated sequences of 7 housekeeping gene fragments of representative *C*. *trachomatis* strains. **Concatenated sequences were aligned and analyzed in MEGA 7**. Phylogenetic tree was constructed using the UMPGA hierarchical clustering method model. Relevant STs & genotypes including in the clustering analyses are presented in Tables 1 & 2, respectively.

Interestingly, the Saratov CT samples of genovar E were grouped in ST4 only. They differed from E/Bour by two alleles (*oppA* and *gitA*) that resulted in their separation from the ST94 of E/Bour to a different cluster (Fig 4). There were no Saratov strains belonging to the ST of E/Bour, although the *ompA* types of all our strains of genovar E (subtype E1) were identical to this reference strain (Table 1, Fig 2).

The group of mono-infected patients (n = 50) was also examined for STs distribution according to the gender, geographical and ethnic origin (S2 Fig), as well age. Overall, two STs, such as ST4 and ST9 were the most prevalent mono-infections among the patients tested. Both STs were found more often in male, female, urban and Slavic population in comparison with other STs. ST4 was observed more frequently over ST6, ST10 and ST38 (*p*<0.01) in female, but not to ST9 (*p*>0.05). ST4 was occurred more often than ST10 (*p*<0.01), as well as both ST6 and ST38 (*p*<0.05) in urban population. In Slavic individuals, ST4 significantly differed from ST38 (*p*<0.05), ST6 and ST10 (*p*<0.01).

ST4 was also found more often in other groups, such as anonymous, rural and Non-Slavic patients. In male and anonymous patients, ST4 demonstrated no significant difference in distribution than other STs (*p*>0.05). ST4 was more often observed in rural compared to other STs (*p*<0.05), including ST10 and ST38 (*p*<0.01). Further, ST4 was the most prevalent in Non-Slavic population in comparison to ST6 and ST38 (*p*<0.05), ST10 (*p*<0.01), but not to ST9 (*p*>0.05). The appearence of ST9 was statistically unsignificant in all groups (*p*>0.05). The ST4 group (patients average age = 25.3) consisted of the Saratov CT strains of the most frequently occurring genovars E and D (Table 2) that were clearly distinguished in *ompA* typing (Table 1, Fig 2). Nevertheless, the ST4 relevant loci of the representative Saratov CT strains were identical to the reference strains of ST4 of both genovars (Table 2). Similarly ST9 (patients average age = 26.6) was also formed by two separate genovars, namely G and J. The alleles of these strains corresponded to the reference strains of ST9 (Table 2). ST6 (patients average age = 29), ST10 (age was unknown, anonymous) and ST38 (average age = 28.5) were represented by only single genovars, such as K, H and F, respectively (Table 2). Each of them was related to the reference CT strain of the assigned ST.

There was no significant difference in distribution of Group I (average age = 27.2) and Group III (average age = 25.8) in the patients tested (p>0.05).

## Discussion

Overall, the clinical isolates of CT, an obligate intracellular pathogen, have been divided into three biovars comprising 15 genovars, namely trachoma (genovars A-C), urogenital Chlamydial infection (genovars D-K), and lymphogranuloma venereum (L1-L3). These biovars differ in disease manifestation and severity [25]. Initially, this classification was based on the antigenic variations within the major outer membrane protein (MOMP) detectable by the MOMP-based micro immunofluorescence test [26]. Currently, the molecular inter- and intra-species differentiation of CT isolates relies on the variability of the four VDs of the *ompA* gene encoding the relevant epitopes of the major outer membrane protein (MOMP) [27]. Although new techniques for the discrimination of *C*. *trachomatis* isolates have been successfully developed, the *ompA*-based genotyping is still the most widely used method for obtaining information on CT genetic variations. There were numerous efforts to find correlates between mutations in the *ompA* gene and relevant changes in phenotype that influence the host immune response, adaptation of the pathogen to diverse host niches, impact on disease severity, and difference in the host susceptibility to CT [25]. Despite the fact that no direct correlation between *ompA* variability and disease severity has been established [11,28] intensive testing of CT isolates during the last decades demonstrated a clear difference in the *ompA*-based genovar distribution worldwide among different ethnicity groups, including Europe, Americas, Africa and Russia [5,12–17]. This observation may indirectly point towards the existence of selectivity of certain CT genovars with respect to different regions and communities. Thus, there were no cases of trachoma registered in Russia since late 1940s which resulted in the lack of detection of CT isolates of the A-C serovars [29–30]. The cases of lymphogranuloma venereum (serovars L1-L3) are also extremely rare, thus a strong prevalence of genital CT genovars (D-K) in this country [5] can be seen. Nevertheless, little is known about the distribution of CT genovars within Russian Regions.

In this study, the prevalence and CT genovar and STs distribution was carefully investigated in a cohort of patients in the Saratov Region, which is one of the 46 large regions of Russian Federation. This region is close to East (Belarus, Ukraine, Moldova) and South (Caucasus Region) European regions on the southwestern border and Kazakhstan on the southeastern border. Overall, this crossroad location between Europe and Asia dictates a multi-ethnic population and high migration area that may explain the relatively high prevalence of chlamydial infection (72.1 per 100,000 people) [31]. This specific geographic setting provided an opportunity to identify the pattern of representative genovars and compare it with the worldwide distribution of most prevalent strains, such as D, E, F and G together with three other genovars, H, J and K, typical for chlamydial genital infection [5,7,12–20,23,32–34]. In contrast to previous surveys from other countries, which revealed that the heterosexual population was infected with the dominating genovars D, E, G, or E alone, or E followed by F [7,15,33], the Saratov Region had a strikingly low representation of the genovar D with the prevalent domination of genovar E followed by G and F (Fig 1A). Similar CT genovar distribution has been recently observed in Greece in male patients with urethritis [14]. Likewise, it was shown recently that in Slovenia and Kharkiv Region (Ukraine) that genovars E, G and F were the most prevalent, although in a different proportion [13,20].

In addition, there was a significant difference in genovar’s distribution among the assessing population and CT patients of European and American women who were mostly infected with either genovars D or Ia. In our study, women demonstrated almost the same genital genovars, i.e. D, E, F, G, J and K, except Ia as shown for American, European and Russian St. Petersburg female patients [5].

Our data also show a slightly different genovar distribution from that in the Moscow Region [3,4], located in the northwest of the Saratov Region. In Moscow, genovar E was also the most dominant and accounted for about 40% of CT cases followed by G variants; however, genovar K was the third prevalent CT variant in the current research (Fig 1A). Moreover, we found more varieties in the genovar E subtypes, which were represented in the Saratov Region by E1, E2 and E6. The latter subtype contains a characteristic G995A SNP (Table 1), which was first detected in chlamydia patients in Sweden [23]. Nevertheless, both Saratov and Moscow Regions shared G1, G2, and G3 genovars, as well as a rare replacement T1003A existing in the G5 variant (Table 1) [4]. Also both Regions showed the presence of two identical variants of genovar D, D1 and Da2, as well as H2 and F1 subtypes (Table 1) [4]. In contrast to SNP at position 1063 reported by Ikryannikova *et al*. [4] for Moscow Region variants of the serovar K, all our seven CT samples of this genovar were identical to the reference strain K/UW31/Cx [12]. Moreover, our study revealed the presence of the J1 subtype, which was not found in two surveys in the Moscow Region [3,4]. Thus, a comparison of genovar distribution in two geographically close areas, Moscow and Saratov Regions, revealed a marked difference in genotypic prevalence. On the other hand, the observed prevalence of genovars E and F corresponds well with with the previous speculations on possible biological advantages for these genovars [7]. Similar assumptions were made for the second most prevalent genovar G (Fig 1A), which is thought to have an increased ability to overcome the host immune defense and possesses an increased transmission [12,13,17,20,23,33–35]. Unfortunately, we could not compare our data with CT genovar distribution from the bordering regions of Kazakhstan due to the lack of such studies for this country.

We applied MLST technique to estimate the diversity of 46 chlamydia samples from Saratov Region, and found five STs. A comparative analysis of their allele profiles employing seven housekeeping genes (Table 2) together with constructed phylogeny tree from the relevant concatenated sequences (Fig 4) demonstrated the presence of two types of strains belonging to Groups identified early [4,9]: (i) Group I was consisted of ST6, ST9 and ST10 (Fig 3A); (ii) Group III was represented by ST4 and ST38 (Fig 3B). ST4 was found to be a putative founder for ST38 clonal complex [4], while ST6, ST9 and ST10 were the founders for other subgroups that were not present in our set of the samples [4,9]. Also we did not uncover the presence of ST13, which was assigned as putative founder for ST6, ST9 and ST10 [4,9]. Nevertheless, this ST13 together with both ST6 and ST9 were recently found in Moscow Region that is relatively close to us geographically [4].

Surprisingly, in assessing population there were significantly less STs (only five STs in the current survey, Table 2) in comparison with those revealed among 58 CT positive patients from St. Petersburg, another Russian Region, located in the northern part of the country and bordering Europe [5]. In consisting with the recently reported classification based on both SNPs in the *ompA* gene and seven housekeeping genes [5], all CT strains associated with non-invasive urogenital disease were grouped within the Haplotype 2. No strains belonging either to the Haplotype 1, associated with invasive urogenital disease caused by LGV, or the Haplotype 3 connected with trachoma caused by typical A-C serovars were found.

Surprisingly, there were no CT strains associated directly with E/Bour (ST94), although all Saratov CT strains were related to STs of strains that are widely distributed around the globe [13]. Initially, *ompA* typing of CT strains from Saratov Region showed the prevalence of variants identical to E1 subtype of CT with E/Bour as the reference representative [12]. However, our strains of the genovar E were MLST identified as ST4 and were related to the ST4 of another reference strain of this genovar, such as isolate SWEDEN2 (Table 2). The latter seems to be a putative founder for ST94 (Fig 3B). Notably, E/Bour and other *C*. *trachomatis* genovar E1 strains could not be differentiated either by *ompA* typing or MLST analysis based on only five housekeeping genes typing [11]. In contrast the seven genes MLST used by us was able to discriminate such cases. This finding could be very useful for molecular epidemiology of CT to know better the population genetic structure. Furthermore, understanding the diversity of CT strains circulating in different regions of the world should be helpful in evaluation of association between CT genotype and the disease. Nevertheless, with the massive sequencing capabilities available, it is becoming increasingly important to connect the knowledge of the global diversification of CT with clinical data.

We also demonstrated three strong trends for the contingent of CT-positive patients in our Region: (i) descending age, (ii) prevalence of females compared with males and (iii) a high frequency (18%) of mixed or co-infection (Fig 1A) that slightly surpassed a 15% range reported in other parts of the world [18,19,32,36,37]. A previous survey in 2005–2007 in both Russia and Saratov Region demonstrated a reversal of statistics with older men as the predominantly infected CT-positive patients [38], which is also similar to data found in other international reports [13,15]. Prevalence of female patients under 30 years old with a clear tendency to further decrease was observed in the current study, which corresponded to the global trend [16,17,33]. The detection of mixed infection is important for understanding *C*. *trachomatis* evolution because of the competitive potential each genovar has in pathogenesis, as well as to increase the quality of Chlamydia diagnostics, partner notification and transmission.

## Conclusions

Thus, data obtained in this study represent a first report on monitoring the basic trends in CT genovar and STs prevalence in the Saratov Region of Russia in order to improve local and national STI services. Our future research will extend this investigation by describing trends in a larger population, both inside and outside of the Saratov Region to clarify some aspects for the actual application of *C*. *trachomatis* genotype analysis for disease control and prevention. Ideally, genotyping analysis should be connected with clinical data in both symptomatic and asymptomatic patients, as well as in different groups including adolescens, pregnants, infants, sexual minorities, etc. Future perspective is also connected with unraveling the genetic variability of both host and pathogen in parallel.

## Supporting information

S1 Table Distribution of genovars in CT-positive chlamydia patients. (DOC)

S1 Fig Distribution of the genovars based on *ompA* diversity in CT-positive patients of 12 different ethnic groups (%) including Russians, Tatars, Jews, Kazakhs, Ukrainians, Caucasians, Byelorussians, Germans, Kyrgyzs, Koreans, Mordovians and Moldavians. (DOC)

S2 Fig Distribution of sequence types (STs) in CT-positive patients with mono-infection according to their gender, place of residence, and ethnic origin. The anonymous patients were included as well. The analysis was performed by using 95% confidence interval (95% CI). Statistically significant differences are indicated by (*p*<0.05). Slavic cohort included Russians, Byelorussians and Ukrainians, Non-Slavic cohort was presented by Caucasians, Jews, Kyrgyzs, Koreans, Moldavians, Germans, Mordovians, and anonymous.
(TIF)
