# Supplementary material for: Urogenital Chlamydia trachomatis multilocus sequence types and genovar distribution in chlamydia infected patients in a multi-ethnic region of Saratov, Russia
Source: PLoS One. 2018 Apr 11;13(4):e0195386. doi: 10.1371/journal.pone.0195386 (PMC5895025; doi:10.1371/journal.pone.0195386)
Supplement: S1 Table — (DOC) [file pone.0195386.s001.doc]

**Table 1. Distribution of genovars in CT-positive Chlamydia patients**

| Genotype | Total  N=61  patients tested (%) | Male  N=12 (%) | Female  N=42  (%) | Ano-nymous  N=7 (%) | Average age | | Total average  age **^*^**  26.4 | Populations | | Ethnic group/Nationality for CT-positive patients | | | |
| --- | --- | --- | --- | --- | --- | --- | --- | --- | --- | --- | --- | --- | --- |
|  |  |  |  |  | Male | Female |  | Urban resi-dents (%) | Rural residents (%) |  |  |  |  |
|  |  |  |  |  |  |  |  |  |  | Slavic **^†^**  (%) | non-Slavic | | |
|  |  |  |  |  |  |  |  |  |  |  | Kazakhs  (%) | Tatars  (%) | Others **^‡^**  (%) |
| Single | 50 (82.0) | 10 (83.3) | 33 (78.6) | 7 (100) | 30.4 | 24.6 | 26 | 35 (83.3) | 15 (78.9) | 26 (78.8) | 2 (50) | 5 (71.4) | 17 (100) |
| D | 4 (8) | 1 (10) | 3 (9.1) | 0 | 27 | 23.7 | 24.5 | 3 (8.6) | 1 (6.7) | 4 (15.4) | 0 | 0 | 0 |
| E | 24(48) | 4 (40) | 16 (48.4) | 4 (57.1) | 27.3 | 24.3 | 24.9 | 14 (40) | 10 (66.7) | 11 (42.3) | 2 (50) | 1 (20) | 10 (58.7) |
| F | 5 (10) | 1 (10) | 3 (9.1) | 1 (14.3) | 25 | 29.7 | 28.5 | 5 (14.3) | 0 | 3 (11.5) | 0 | 0 | 2 (11.8) |
| G | 9 (18) | 3 (30) | 6 (18.2) | 0 | 32.7 | 24.2 | 27 | 8 (22.9) | 1 (6.7) | 6 (23.1) | 0 | 1 (20) | 2 (11.8) |
| H | 1 (2) | 0 | 0 | 1 (14.3) | 0 | 0 | 0 | 1 (2.9) | 0 | 0 | 0 | 0 | 1 (5.9) |
| J | 2 (4) | 0 | 2 (6.1) | 0 | 0 | 24.5 | 24.5 | 1 (2.9) | 1 (6.7) | 0 | 0 | 1 (20) | 1 (5.9) |
| K | 5 (10) | 1 (10) | 3 (9.1) | 1 (14.3) | 45 | 23.6 | 29.3 | 3 (8.6) | 2 (13.3) | 2 (7.7) | 0 | 2 (40) | 1 (5.9) |
| Multiple | 11 (18.0) | 2 16.7) | 9 (21.4) | 0 | 32 | 26 | 27.1 | 7 (16.7) | 4 (21.1) | 7 (21.2) | 2 (50) | 2 (28.6) | 0 |
| D+F | 1 (9.1) | 1 (50) | 0 | 0 | 36 | 0 | 36 | 1 (14.3) | 0 | 0 | 0 | 1 (50) | 0 |
| E+F | 1 (9.1) | 1 (50) | 0 | 0 | 28 | 0 | 28 | 0 | 1 (25) | 0 | 0 | 1 (50) | 0 |
| E+G | 3 (27.3) | 0 | 3 (33.4) | 0 | 0 | 23.3 | 23.3 | 1 (14.3) | 2 (50) | 3 (42.9) | 0 | 0 | 0 |
| E+K | 1 (9.1) | 0 | 1(11.1) | 0 | 0 | 22 | 22 | 1 (14.3) | 0 | 0 | 1 (50) | 0 | 0 |
| F+G | 2 (18.2) | 0 | 2 (22.2) | 0 | 0 | 23 | 23 | 1 (14.3) | 1 (25) | 2 (28.6) | 0 | 0 | 0 |
| F+K | 1 (9.1) | 0 | 1 (11.1) | 0 | 0 | 35 | 35 | 1 (14.3) | 0 | 0 | 1 (50) | 0 | 0 |
| E+H+G | 1 (9.1) | 0 | 1 (11.1) | 0 | 0 | 40 | 40 | 1 (14.3) | 0 | 1 (14.3) | 0 | 0 | 0 |
| E+J+G | 1 (9.1) | 0 | 1 (11.1) | 0 | 0 | 21 | 21 | 1 (14.3) | 0 | 1 (14.3) | 0 | 0 | 0 |
| Total | 61 (100) |  |  |  |  |  |  | 42 (68.9) | 19 (31.1) | 33 (54.1) | 4 (6.5) | 7 (11.5) | 17 (27.9) |

***** from 54 none-anonymous CT-patients

**†** East Slavic cohort included Russians, Byelorussians and Ukrainians

**‡** Caucasians, Jews, Kyrgyzs, Koreans, Moldavians, Germans, Mordovians, anonymous
