# Supplementary figures and images for: Urogenital Chlamydia trachomatis multilocus sequence types and genovar distribution in chlamydia infected patients in a multi-ethnic region of Saratov, Russia
Source: PLoS One. 2018 Apr 11;13(4):e0195386. doi: 10.1371/journal.pone.0195386 (PMC5895025; doi:10.1371/journal.pone.0195386)

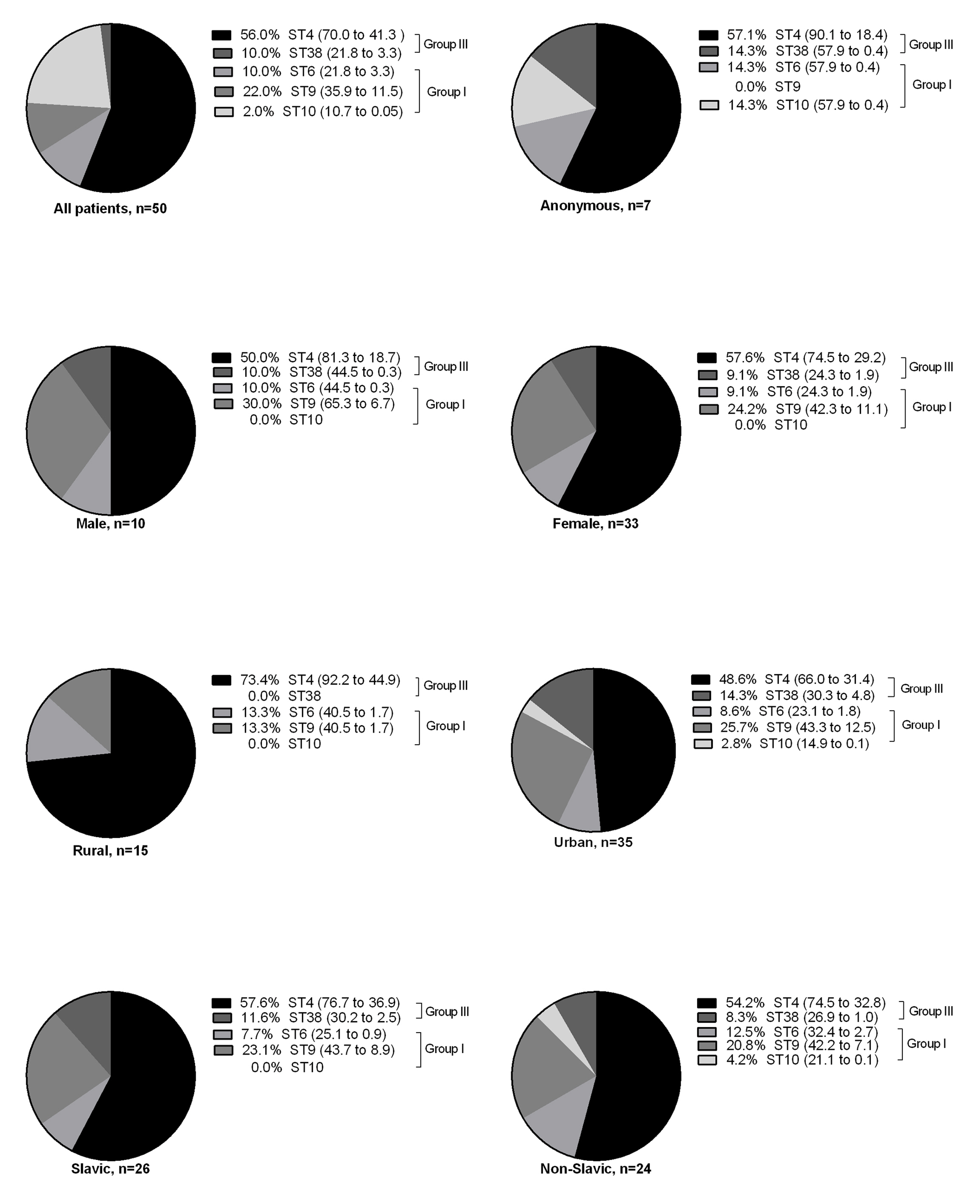

Supplement: S2 Fig — The anonymous patients were included as well. The analysis was performed by using 95% confidence interval (95% CI). Statistically significant differences are indicated by (p<0.05). Slavic cohort included Russians, Byelorussians and Ukrainians, Non-Slavic cohort was presented by Caucasians, Jews, Kyrgyzs, Koreans, Moldavians, Germans, Mordovians, and anonymous. (TIF) [file pone.0195386.s003.tif]
