# Supplementary material for: Urogenital Chlamydia trachomatis multilocus sequence types and genovar distribution in chlamydia infected patients in a multi-ethnic region of Saratov, Russia
Source: PLoS One. 2018 Apr 11;13(4):e0195386. doi: 10.1371/journal.pone.0195386 (PMC5895025; doi:10.1371/journal.pone.0195386)
Supplement: Supplementary file 3 [file pone.0195386.s002.doc]

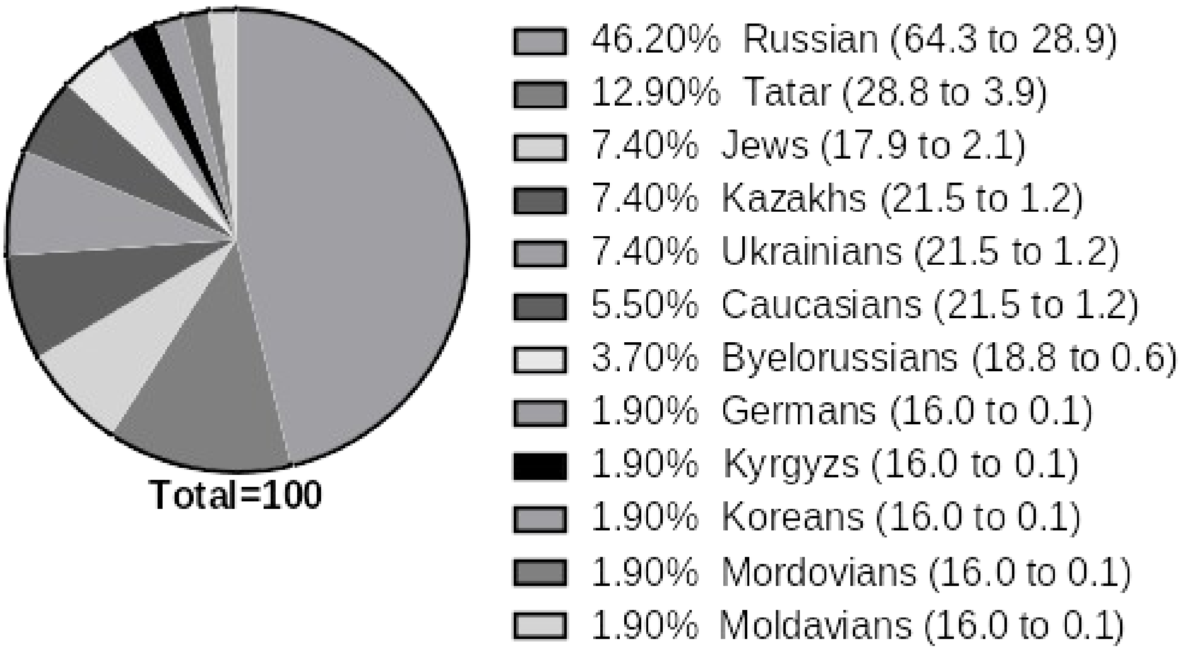

Total=100
46.20% Russian (64.3 to 28.9)
12.90% Tatar (28.8 to 3.9)
7.40% Jews (17.9 to 2.1)
7.40% Kazakhs (21.5 to 1.2)
7.40% Ukrainians (21.5 to 1.2)
5.50% Caucasians (21.5 to 1.2)
3.70% Byelorussians (18.8 to 0.6)
1.90% Germans (16.0 to 0.1)
1.90% Kyrgyzs (16.0 to 0.1)
1.90% Koreans (16.0 to 0.1)
1.90% Mordovians (16.0 to 0.1)
1.90% Moldavians (16.0 to 0.1)
